# Partial Solution of Callus from Low Living

**Published:** 1844-11-02

**Authors:** 


### PARTIAL SOLUTION OF CALLUS FROM LOW LIVING.

John A——, aged 16, a labouring lad, admitted into the London hospital under Mr. Scott, Sept. 14. He complained of considerable pain in the right thigh, increased materially during progression. He moved very slowly, with a faultering gait and great caution.

On examining the thigh it presented a considerable bulging forwards, a little above the centre, in which situation the medium that had united the two originally separated portions of the bone had become partially absorbed, the upper and lower part of the femur admitting of slight but obvious independent movement when grasped by the hands: he appeared thin and weak, the features being pale, and the pulse small and hurried.

About two years ago, he was an in-patient, having received a simple fracture of the right thigh, near the upper third. He was under treatment about nine weeks, and when he was discharged the limb was perfectly firm, and he could walk with facility. From the hospital he became the inmate of a neighbouring union, where he remained till the period of his second admission. His diet during this time consisted of five ounces of meat three times in the week, soup three times, and bread and cheese once; and for about six or eight months, at different periods, he had by request of the medical attendant, half a pint of porter daily. The symptoms of the uniting medium giving way were first indicated by local pain, afterwards by imperfection of gait, and increased prominence of the thigh bone, forwards and outwards, at the original seat of fracture. The pain commenced about three months before he entered the hospital.—He was placed on full diet, by which he was allowed fifteen ounces of meat in the week more than he received in the workhouse, and a quantity of soup equal in volume and strength to what he drank there; he took, also, a little decoction of bark, and half a drachm of sesquioxide of iron, three times in the day, and an occasional rhubarb purge. The recumbent position was strictly enjoined. Having been under this treatment for seven weeks he left the hospital, firm union of the bone having resulted.—*London Lancet.*

